# Supplementary material for: A computational framework for physics-informed symbolic regression with straightforward integration of domain knowledge
Source: Sci Rep. 2023 Jan 23;13:1249. doi: 10.1038/s41598-023-28328-2 (PMC9870915; doi:10.1038/s41598-023-28328-2)
Supplement: Supplementary file 1 — Supplementary Information. [file 41598_2023_28328_MOESM1_ESM.pdf]

A Computational Framework For Physics-Informed Symbolic Regression with Straightforward Integration of Domain Knowledge

Liron Simon[1,*], Alex Liberzon[1], Teddy Lazebnik[2]

1 Turbulence Structure Laboratory, School of Mechanical Engineering, Tel Aviv University, Israel
2 Department of Cancer Biology, Cancer Institute, University College London, UK

*** lirons.gb@gmail.com**

Appendix

Physics behind experiments A-E

We constructed the experiments used in SciMED testing around the problem of a sphere settling in a fluid under various fluid models and simplifying assumptions. The physics of settling spheres can be very simple at low velocities or very viscous fluids, with an analytically solved linear drag force. It can become very difficult when the settling velocities are not negligible, and the drag force becomes a highly non-linear function of the velocity itself. In this part, we draw the parallel between the physics problem and the mathematical description used for the SciMED for clarity. The experiments are constructed similarly to physics experiments in which we measure some physical properties, similar to the case presented in [1], repeating in some sense the famous Leaning Tower of Pisa experiment.

Experiment A

If in the experiment we measure velocity $v(t)$ and acceleration $a(t)$ of the settling sphere versus time, we can present experiment A as the simplest case in which $a = \text{const}$, e.g. by [2]:

$$v = v_0 + a\,t \tag{1}$$

where $v_0 = v(t = 0)$ is the initial velocity of that object, a is its acceleration, and t is the elapsed time from v_0 to v. In this experiment, we provide the system with a table of the measurements of multiple spheres for each the initial velocity, time since the release, and acceleration as features, along with the velocity as a target.

Experiment B-E

For experiments B-E, we formulate our problem slightly differently. Similar to the Leaning Tower of Pisa experiment and [1] we aim to find a law of falling bodies, extending it with an unknown fluid resistance force. We are interested in finding the unknown equation for drag force F_D exerted on a sphere settling at constant velocity V through a viscous fluid. It is common to present the drag force using the non-dimensional drag coefficient C_D:

$$F_D = 0.5\,C_D\,\rho\,v|v|A \tag{2}$$

where ρ is the fluid density, v is the velocity of the sphere relative to the fluid velocity, $A = \pi d^2/4$ is the cross-sectional area of the sphere, d is the diameter of the sphere, and C_D is the dimensionless drag coefficient [3]. This equation is used for experiment E, assuming the simplest cases of spheres settling in the same direction of gravity (meaning $v|v| = v^2$).

For the rest of the experiments, we also consider the spheres to be of smooth surface, settling with a non-dimensional Reynolds number $Re = Vd/\nu$, where ν is the kinematic viscosity of the fluid, of up to

$Re = 2 \times 10^5$. In this case, the drag coefficient can be expressed as a semi-analytical, semi-empirical function with all non-dimensional terms (including a numerical constant 0.4, 24, and others) [3]:

$$C_D = 0.4 + \frac{24}{Re} + \frac{6}{1+\sqrt{Re}}. \tag{3}$$

When the sphere moves at a constant velocity (zero acceleration), the drag balances the buoyancy force (first term on the right-hand side):

$$0 = (\rho_p - \rho) g V - F_D, \tag{4}$$

where ρ_p is the sphere's density, and $V = \pi d^3/6$ is the volume of the sphere. Extracting C_D from Eqs. 3-4, we obtain:

$$C_D = \frac{4}{3}\frac{\rho_p - \rho}{\rho}\frac{gd}{v^2}. \tag{5}$$

For experiment C, we use the dimensional form of the Eq. 5, substituting $g = 9.81$ m s^{-2} and looking for the form of:

$$C_D = 13.08\,\frac{\rho_p - \rho}{\rho}\frac{d}{v^2}. \tag{6}$$

where 13.08 m s^{-2} is a "modified" gravitational acceleration for spheres.

Note that in experiment D, we use again the Eq. 6 to generate the data, but then eliminate the measurements of velocity v before analyzing the data with SciMED.

Experiment B - feature selection part

In experiment B, we use the non-dimensional form of Eq. 5 to emphasize the strength of the dimensional analysis along with the feature selection component of SciMED. Therefore, experiment B aims to a) test nine hypotheses of dimensionless numbers governing the dynamic in a feature selection step and b) discover the function for non-dimensional C_D in Eq. 5. The ground truth based on the domain knowledge is the multiplication of two non-dimensional numbers: $\zeta_1 = (\rho_p - \rho)/(\rho)$ and $\zeta_2 = (gd)/(v^2)$ with a constant non-dimensional prefactor of 4/3.

Dimensional analysis helps to formulate the search for the unknown non-linear function C_D as a function of non-dimensional arguments. Initially, we hypothesize nine plausible dimensionless ratios that may represent some key mechanism for determining C_D. The ratios can be seen as groups of dimensionless parameters, where each parameter in a group represents a different physical relation. Therefore, the feature selection component aims to identify which number in a group is dominant over the others, potentially emphasizing the dominant physical mechanism. Only one non-dimensional parameter per group will participate in the following steps.

We formed nine groups of dimensionless ratios using the physical properties of the problem, i.e., properties of the sphere: d, ρ_p, properties of the fluid: ρ, ν, and a measured quantity, particle velocity v. We deliberately extended the number of groups and the possible variations to probe for the previously unexplored physical mechanisms. Thus, we assume a possible effect related to some natural frequency, N (units of 1/sec). This frequency is typically coupled with a representative time scale, and in some problems, it's called the Froude number. We suggest four such groups, each defined based on different time scale: $v/(dN), g/(vN), (gd)/\nu N), v^2/(\nu N)$. Because we can construct frequency based on various density ratios (particle to fluid, a difference of particle density to fluid density, and so on), we can formulate seven possible combinations that we call for simplicity: N :

$$N_i = \sqrt{\frac{g\,\rho_\alpha}{d\,\rho_\beta}}, \tag{7}$$

where $(\rho_\alpha, \rho_\beta)$ is one of the seven density combinations: $(\rho_p - \rho, \rho_{\text{avg}})$, $(\rho_p - \rho, \rho_p)$, $(\rho_p - \rho, \rho)$, $(\rho_p, \rho_{\text{avg}})$, (ρ_p, ρ), $(\rho, \rho_{\text{avg}})$, (ρ, ρ_p), and $\rho_{\text{avg}} = 0.5(\rho_p + \rho)$.

The other five groups are a) ratios of densities ρ_p/ρ, b) the group of the ratio of density difference to density $(\rho_p - \rho)/\rho$, c) two groups of the combinations gd/v^2, d) $\nu g/v^3$, and e) the single-element group of the

Reynolds number Re that in this simple case of a single length scale, d, and a single velocity scale v, cannot get another representation.

Fig.2 represents the nine groups above on the left-hand side. The first four groups of Froude-like numbers contain seven plausible non-dimensional parameters from which only one is chosen to proceed to the SR component. The other five groups contain only one non-dimensional parameter, which proceeds to the SR without a selection process. During the SR, the remaining nine non-dimensional parameters are reduced to the two that construct the equation and emphasize the contribution of each group to the physical problem.

Comparing the relative contribution to the explainability of the target of the nine parameters selected by the feature selection component (the middle column in Fig.2) may lead to additional insight. In more complex problems, the non-dimensional parameter selected from the group can indicate different physical mechanisms. For instance, in the drag force Eq. 3, one could study in detail how fluid resistance force changes when the Reynolds number increases and what happens when C_D varies from a constant 0.4 to $24/Re$ and then from linear relation to a non-linear $6/(1+\sqrt{Re})$. This competition of the nine groups is provided by a feature importance graph produced by the AutoML component, as seen in Fig.1.

References

1. Brian M. de Silva, David M. Higdon, Steven L. Brunton, and J. Nathan Kutz. Discovery of physics from data: Universal laws and discrepancies. *Frontiers in Artificial Intelligence*, 3:25, 2020.

2. G Elert. The physics hypertextbook, equations of motion.

3. Frank M White and Joseph Majdalani. *Viscous fluid flow*, volume 3. McGraw-Hill New York, 2006.
